# The role of mast cells and fibre type in ischaemia reperfusion injury of murine skeletal muscles

**DOI:** 10.1186/1476-9255-1-2

**Published:** 2004-09-27

**Authors:** Susan K Bortolotto, Wayne A Morrison, Aurora Messina

**Affiliations:** 1 Bernard O'Brien Institute of Microsurgery, Fitzroy Street, Fitzroy, AUSTRALIA; 2 Department of Surgery, University of Melbourne, St. Vincent's Hospital, Melbourne, Victoria, AUSTRALIA

## Abstract

**Background:**

Ischaemia reperfusion (IR) injury of skeletal muscle, is a significant cause of morbidity following trauma and surgical procedures, in which muscle fibre types exhibit different susceptibilities. The relative degree of mast cell mediated injury, within different muscle types, is not known.

**Methods:**

In this study we compared susceptibility of the fast-twitch, extensor digitorum longus (EDL), mixed fast/slow-twitch gastrocnemius and the predominately slow-twitch soleus, muscles to ischemia reperfusion (IR) injury in four groups of mice that harbour different mast cell densities; C57/DBA mast cell depleted (W^f^/W^f^), their heterozygous (W^f^/+) and normal littermates (+/+) and control C57BL/6 mice. We determined whether susceptibility to IR injury is associated with mast cell content and/or fibre type and/or mouse strain. In experimental groups, the hind limbs of mice were subjected to 70 minutes warm tourniquet ischemia, followed by 24 h reperfusion, and the muscle viability was assessed on fresh whole-mount slices by the nitroblue tetrazolium (NBT) histochemical assay.

**Results:**

Viability was remarkably higher in the W^f^/W^f ^strain irrespective of muscle type. With respect to muscle type, the predominately slow-twitch soleus muscle was significantly more resistant to IR injury than gastrocnemius and the EDL muscles in all groups. Mast cell density was inversely correlated to muscle viability in all types of muscle.

**Conclusion:**

These results show that in skeletal muscle, IR injury is dependent upon both the presence of mast cells and on fibre type and suggest that a combination of preventative therapies may need to be implemented to optimally protect muscles from IR injury.

## Background

Ischemia reperfusion injury is a widespread phenomenon that affects all muscle tissues [1,2]. It is a significant cause of morbidity following injury especially to limb blood vessels with resultant muscle necrosis, fibrosis and joint contracture (Volkmann's contracture). Of the muscle involved some are slow-twitch 'red' fibre type predominately designed for sustained isometric contraction to stabilise joints while the other fast-twitch 'white' fibre type muscles act with speed and dexterity such as the lumbricals and flexor digitorum profundi. In the leg the soleus is predominately slow-twitch, the extensor digitorum longus (EDL) fast-twitch and the gastrocnemius is mixed slow/fast-twitch fibre type.

Mast cells were first implicated in IR injury of skeletal muscle in studies from our laboratory [3]. Initial experiments in the gastrocnemius muscle, resulted in resistance to IR injury in mast cell depleted (W^f^/W^f^) mice [3,4]. More recently we demonstrated that re-engraftment of mast cells into W^f^/W^f ^mice restores susceptibility to IR injury, thus proving that mast cells play a pivotal role in IR injury to skeletal muscle [5]. Our IR injury model consists of 70 minutes tourniquet hind limb ischaemia followed by 24 h reperfusion. Unlike other models [6], the extended reperfusion period permits full manifestation of the reperfusion injury. In order to determine the usefulness of therapies against mast cells, it is important to know the degree to which mast cells are involved in IR injury of other skeletal muscle fibre types. In this study we selected skeletal muscles, representative of slow-twitch (soleus), slow/fast-twitch (gastrocnemius) and fast-twitch (EDL) types, and compared their susceptibility to IR injury in four genotypically different sets of mice that harbour different mast cell densities in their skeletal muscle. These were the C57/DBA mast cell depleted (W^f^/W^f^) mice, their heterozygous (W^f^/+) and normal littermates (+/+) and control C57BL/6 mice.

## Methods

### Animals

Mast cell depleted mice, W^f^/W^f^, (C57BL/6W^f ^× DBA/2W^f^)F_1_; wild-type littermate, +/+, (C57BL/6 × DBA/2)F_1_; and heterozygous littermate, W^f^/+, (C57BL/6 × DBA/2W^f ^or C57BL/6W^f ^× DBA/2)F_1 _hybrids were purchased from Flinders Medical Centre (Bedford Park, South Australia) aged 6–10 wk (18–25 g). Each genotype was clearly identified by coat colour. C57BL/6 × C57BL/6 (C57BL/6) mice were purchased from Animal Resources Centre, Perth, Western Australia aged 10 wk (25–30 g). A C57BL/6 strain mouse group was included as an additional control to the C57/DBA strain, to test for strain differences in susceptibility to IR injury. This was important, as the C57BL/6 is the most commonly used mouse strain. There was no significant difference in the data between male and females so the results were pooled. Mice were given food and water ad libitum, and housed with a 12 h day/night cycle. The Animal Ethics Guidelines outlined by St. Vincent's Hospital and National Health & Medical Research Council were adhered to in all experiments.

### Ischemia-Reperfusion injury

Mice were anaesthetized by intraperitoneal (i.p.) injection of 4% chloral hydrate (0.1 ml/10 g body weight), followed by i.p. injection of the analgesic carprofen to minimise postoperative pain. Tourniquet warm ischemia was induced by using 2 × size 8 rubber bands as previously described [5]. During the 70 min ischemia, a needle thermistor probe was inserted subcutaneously in the right leg, and the hind limb temperature was monitored and maintained at 36 ± 1°C. After the ischemia period, the bands were removed and the mice allowed to recover. After 24 h reperfusion the mice were re-anaesthetized and the gastrocnemius, soleus and EDL muscles were carefully removed from both treated and contralateral sides and weighed, before the mouse was sacrificed.

Age matched (12–24 wk, 25–35 g) male and female mice from each genotype (W^f^/W^f^, W^f^/+, +/+ and C57BL/6) were grouped into the same sex, age and strain and underwent warm ischemia at the same time. A minimum of four mice (n = 4) from each of the four genotypes was used.

### NBT Assay

Nitro Blue Tetrazolium (NBT) assay was used to determine muscle viability in fresh whole mounts slices as previously described [5]. Both sides of each muscle slice were post-fixed in 10% buffered formal saline (BFS) and analysed under a dissecting microscope for viable tissue, which was identified by its blue reaction product. The percentage of viable tissue in treated muscle was determined by standard point counting technique [7] and was expressed as a percentage of viable tissue in the contralateral control. Finally, the mean percent viable tissue of treated versus contralateral muscles was calculated for each group where n = number of mice.

### Histology

For histological analysis, muscle slices were immersion fixed for 24 h in 10% BFS, washed in 0.1 M phosphate buffered saline (PBS) and processed into paraffin. Five micron sections were cut, dewaxed and stained with Haematoxylin and Eosin (H&E) for general analysis [5].

### Mast Cell Staining

Mast cells were selectively stained by routine toluidine blue [8] and chloroacetate esterase (CAE) methods [9].

### Mast Cell Density

An overview of mast cell content in the four groups of mice tested was obtained by counting a minimum of 100 mast cell profiles in tongue, skin and heart as well as skeletal muscle. Using 100 X magnification all mast cell profiles that fell within a grid area of 1.35 mm^2 ^but did not touch the right hand and bottom side boundaries were counted and the data expressed as mast cells/mm^2^. There was no difference in the toluidine blue or CAE labelled mast cell profile numbers in comparable tissue sections (data not shown), hence the CAE technique was subsequently used in preference to toluidine blue.

### Statistics

Statistical analyses were performed using SPSS software (Statistical Package for the Social Sciences, version 11.5). All results are expressed as means ± standard error of the mean (SEM) of grouped data where n = number of mice/group. For comparison between groups, means were analysed using univariate analysis of variance. Pearson's correlation was used to test the correlation between tissue viability and mast cell profile counts. A probability level of p < 0.05 was taken to indicate statistical significance.

## Results

### Morphologic appearance of labelled mast cells

After CAE or toluidine blue staining, mast cells were easily distinguished from other cells, by their red or purple stained cytoplasmic granules respectively. In general, they were intact but varied in size, and were predominantly located near nerves and blood vessels as reported by others [10].

### Histological appearance of skeletal muscle before and after IR injury

**Figure 1:**
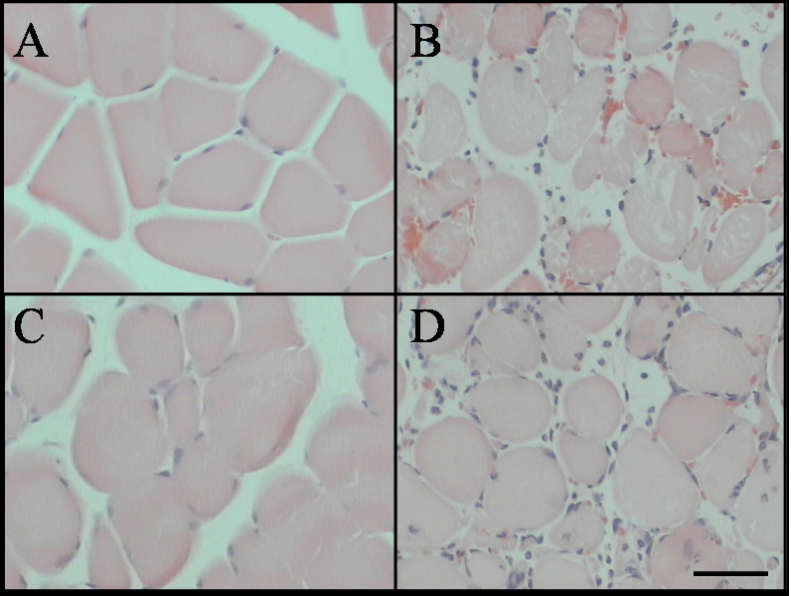
Gastrocnemius muscle stained with Haematoxylin and Eosin. Control (A & C) and IR treated hind limbs (B & D) from littermate control (A & B) and W^f^/W^f ^(C & D) mice. Scale bar = 50µm.

Prior to injury, the morphologic appearance of transverse muscle sections from littermate controls and W^f^/W^f ^mice was similar (see Figure 1A and 1C). As expected, the muscle fibres were nucleated, intact and arranged in groups. In longitudinal sections (not shown), the muscle striations were clearly evident, thus demonstrating viable fibres. After IR injury, muscles from normal littermate mice (Figure 1B) were infiltrated by numerous inflammatory cells that were often observed invading the muscle fibres. Many fibres were fragmented and appeared moth eaten, while other fibres were condensed and shrunken. At high magnification (not shown), the sarcomeric pattern was not visible in the majority of the fibres and many fibres did not contain nuclei. In contrast, a large proportion of fibres from W^f^/W^f ^mice were intact, nucleated, stained amorphously and comparable in size to controls. Surprisingly, this tissue also contained a cellular inflammatory exudate that was largely confined to the interstitial area (Fig. 1D).

### Mast cell density of different organs

**Figure 2:**
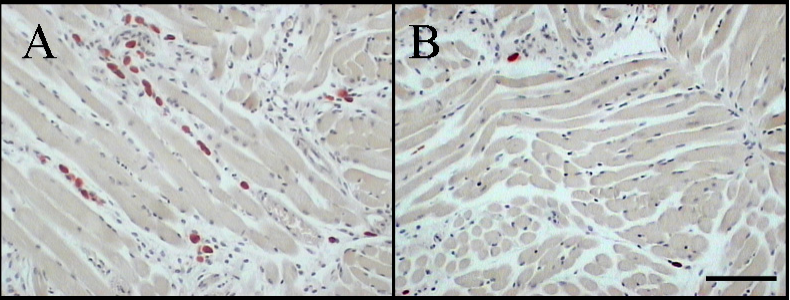
An example of histological sections of tongue stained with chloroacetate esterase for identification of mast cells in littermate controls (A) and W^f^/W^f ^(B) mice. Note mast cells appear as brilliant red colour. Scale bar = 100µm.

The density of CAE stained mast cells varied between tissues and between mouse strains (see Table 1). In mast cell replete mice (C57BL/6, +/+, W^f^/+), the tongue and skin had a consistently high mast cell density ranging from 31.3 to 49.9 mast cell profiles/mm^2^. Cardiac muscle had significantly fewer mast cells ranging from 0.3 to 2.3 mast cell profiles/ mm^2^. In W^f^/W^f ^mice, the mast cell density was markedly reduced in both tongue (1.95 ± 0.42) (Figure 2) and skin (6.73 ± 3.27). No mast cells were observed in W^f^/W^f ^cardiac muscle following screening of a large number of sections (20 fields at X 200 magnification for each mouse).

**Table 1:** Mast cell profile number of tissues from different mouse strains.

Animal strain	Heart	Tongue	Skin
C57BL/6	1.52 ± 0.42	40.31 ± 3.25	31.28 ± 1.89
+/+	2.27 ± 0.39	43.36 ± 3.79	38.73 ± 9.60
W^f^/+	0.29 ± 0.10	37.15 ± 2.23	49.90 ± 10.10
W^f^/W^f^	ND	1.95 ± 0.42	6.73 ± 3.27

Mean ± SEM (n = 4); ND-not detected

### Mast cell density of skeletal muscles

Table 2 shows the mast cell density of each skeletal muscle type in four different groups of mice. In general the slow twitch soleus muscle had almost twice the mast cell density of both the EDL and gastrocnemius muscles (p < 0.05). There were no mast cells in the C57/DBA W^f^/W^f ^skeletal muscles as expected. However, there was twice the density of mast cells in their normal compared to their heterozygous littermates, suggesting a c-kit gene dosage effect on mast cell density (p < 0.05). There were significantly fewer mast cells in the C57/C57 compared with C57/DBA littermate control mice (p < 0.05) but no difference in the mast cell density of C57/C57 compared with C57/DBA heterozygous mice.

**Table 2:** Mast cell profile number of skeletal muscles from different mouse strains.

Animal strain	Soleus	Gastrocnemius	EDL
C57BL/6	2.1 ± 0.4	1.0 ± 0.2	1.3 ± 0.2
+/+	3.7 ± 0.6	1.8 ± 0.3	2.3 ± 0.3
W^f^/+	2.2 ± 0.4	1.0 ± 0.1	1.6 ± 0.2
W^f^/W^f^	ND	0.0 ± 0.0*	ND

Mean ± SEM (n = 4); ND-not detected

*only one mast cell was detected from all tissue examined

### Skeletal Muscle Viability after IR

**Figure 3:**
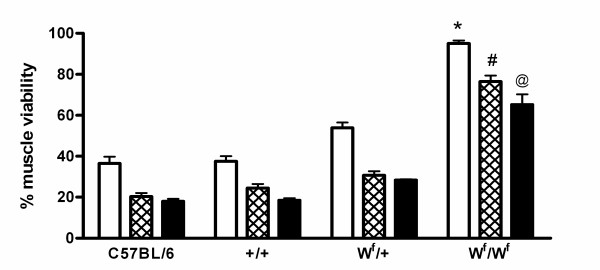
Muscle viability (% contralateral control) as assessed by NBT assay in the soleus (white bars), gastrocnemius (hatched bars) and EDL (black bars) muscles. All values are mean ± SEM, n = 4. * P < 0.05 Significantly different to C57BL/6, +/+ and W^f^/+ mice. # P < 0.001 Significantly different to C57BL/6, +/+ and W^f^/+ mice. @ P < 0.001 Significantly different to C57BL/6, +/+ and W^f^/+ mice.

Skeletal muscles from W^f^/W^f ^mice were significantly more resistant to tourniquet induced warm ischaemia/reperfusion injury, as assessed by NBT assay, compared to the other 3 mouse strains irrespective of muscle type (Fig 3). In the W^f^/W^f ^mice the soleus muscle was a remarkable 95% viable after IR. With respect to the muscle types, the slow-twitch soleus muscle sustained significantly less (P < 0.05) IR injury compared to the slow/fast gastrocnemius and the fast EDL muscles in each group. The gastrocnemius and EDL muscles showed a similar degree of injury in all groups.

### Correlation between mast cell density and viability

There was an inverse correlation (Pearson's correlation factor 0.043) between mast cell density and muscle viability for each muscle type in C57/DBA mice.

### Mouse strain susceptibility

The viability of muscles from C57/DBA heterozygous mice was significantly greater than the C57/C57 controls (p < 0.05) even though the mast cell density was the same.

There was no difference in the viability of muscles from the C57/C57 controls compared with C57/DBA littermate controls even though there was double the mast cell density in the latter muscles.

## Discussion

In this study a tourniquet was placed high up on the thigh to induce a short warm (36°C) ischaemia of 70 minutes duration followed by a long reperfusion period of 24 h, in order to assess the impact of reperfusion injury on different skeletal muscles and the degree to which mast cells mediate this injury. This differs from other studies where an extended ischaemia and short reperfusion period is used to study the early effects of ischaemia on skeletal muscle. Using this approach, we show that mast cells contribute to ischaemia reperfusion injury of fast-, mixed fast/slow- and slow-twitch muscle types. Viability of these muscles was inversely correlated with mast cell density and all muscles exhibited a remarkable resistance to IR injury in mast cell depleted mice. In the absence of mast cells, the predominately slow-twitch oxidative soleus muscle was more resistant to IR than the fast/slow-twitch gastrocnemius and the fast-twitch EDL muscles. We also demonstrate that muscle fibre type, and mouse strain independently, determined susceptibility to IR injury.

The susceptibility of different skeletal muscle types to ischaemia is hypothesized to relate to their different metabolic disposition, but data regarding this is conflicting. Skeletal muscles in mice are composed of two main distinct fibre types. In general, slow-twitch fibres have a high oxidative enzyme activity, high capillary density and increased numbers of mitochondria; in contrast fast-twitch fibres have high glycolytic enzyme activity, low capillary density and few mitochondria. It has been suggested that fast-twitch muscles display a greater resistance to ischaemia than slow-twitch muscles because of their greater potential to maintain ATP levels during ischaemia [11]. Alternatively, it has been proposed that the greater accumulation of anaerobic metabolites during ischaemia in the fast-twitch fibres, compared to the slow-twitch fibres, give rise to oxygen free radicals during reperfusion that makes them more susceptible to injury. In our study, the predominately slow-twitch soleus muscle was consistently more resistant to IR injury than the slow/fast- and fast-twitch muscles. Data from other studies are difficult to compare because of the wide variety of IR models in use. In particular, there is a great deal of variation in the period of ischaemia, the muscle temperature during ischaemia and the period of reperfusion allowed for manifestation of the reperfusion injury. Idstrom [12] utilised a period of 2, 4 and 6 h cold 25°C hind limb ischaemia in rats and one hour reperfusion to measure damage and recovery of adenine nucleotides. Consistant with our data, he showed that the fast-twitch tibialis muscles displayed a faster degradation rate and slower recovery of these molecules than the slow-twitch soleus. This was attributed to differences in the regulation of enzymes during ischaemia and differences in blood flow during reperfusion. Woitaske *et al. *[13] used 3 h of hind limb ischaemia at a unknown temperature and up to 14 days reperfusion in mice. The soleus was less injured and recovered function and mass more quickly than the EDL muscle over this period. In contrast to our data, other groups [6] have shown that after a lengthy ischaemia time of 3 h and a short reperfusion (2 h) fast-twitch muscles are more resistant to injury than slow-twitch muscles. Other workers report variable results. Rácz *et al. *[14] showed that slow-twitch muscles were more severely damaged after 1 hour of ischaemia however the fast-twitch muscle was more damaged after 2 h. Sternbergh [15] used an in vitro model of 120 min ischaemia and 55 min reperfusion at 37°C in rat hind limb. The slow-twitch soleus and fast-twitch plantaris showed similar degrees of injury whereas the fast-twitch tibialis was uninjured. He concluded that muscle fibre type does not predict injury. In Carvalho's study [11] the fast-twitch muscle was better able to contract during the first 45–60 minutes of ischaemia but both fast- and slow-twitch muscles contracted to equal degrees thereafter.

We have recently shown conclusively that mast cells play a pivotal role in IR injury of murine soleus, EDL and gastrocnemius muscles [5]. W^f^/W^f ^IR resistant mice were engrafted with bone marrow derived mast cells (BMMC) from their normal littermates, and their hind limbs underwent IR injury 12 weeks later. The proportion of viable muscle fibres in engrafted mice was significantly reduced, back to the levels observed in their IR susceptible littermates. Thus, engraftment of BMMC into W^f^/W^f ^mice restores the susceptibility of skeletal muscles to IR injury irrespective of the other abnormalities in these mice. The role of mast cells has not been considered when investigating the susceptibility of different muscle fibre types.

In the current study, mast cell density was inversely correlated with survival for all muscle types. In all four strains examined, muscles from the W^f^/W^f ^mice had a significantly greater viability. In particular, the soleus muscle viability was 95% in the mast cell depleted mice indicating that a large amount of injury was mast cell mediated. The gastrocnemius and EDL muscle viability was 76% and 65% respectively in the absence of mast cells, indicating that other factors, possibly related to muscle type contribute to the IR injury. The demonstration that muscles from the C57/C57 and C57/DBA strains of mice were equally affected by IR, even though the latter contained twice the density of mast cells, indicate that there is also a genetic component to IR injury.

Our data would support the hypothesis that there is a base line level of susceptibility to ischaemia induced injury that can be attributed to mouse strain and muscle fibre type. Mast cells independently exacerbate IR injury during a clinically relevant extended reperfusion. These findings predict that mast cell therapies would be beneficial across different muscle types and that further protection can be tailored to specific muscle types. It is clear that mast cell depleted mice are the desirable model to study the effects of IR on muscle fibre type. Alternatively, the soleus is the most suitable muscle to study the role of mast cells since it has the least fibre type component effect.

## Abbreviations

BFS, buffered formal saline; CAE, chlororacetate esterase; EDL, extensor digitorum longus; IR, ischaemia reperfusion; NBT, nitro blue tetrazolium; NOS II, nitric oxide synthase II; W^f^/W^f^, mast cell depleted mice; W/W^v^, mast cell deficient mice.

## Authors' contributions

SKB performed animal experimentation, muscle viability studies, mast cell densities and drafted the original manuscript. WAM participated in the design of the study. AM participated in design of the study and performed morphological analyses. All authors provided intellectual input, participated in the manuscript preparation and have approved the final manuscript.
